# Embodied Carbon in China’s Export Trade: A Multi Region Input-Output Analysis

**DOI:** 10.3390/ijerph19073894

**Published:** 2022-03-24

**Authors:** Weixin Yang, Hao Gao, Yunpeng Yang, Jiacheng Liao

**Affiliations:** 1 Business School, University of Shanghai for Science and Technology, Shanghai 200093, China; iamywx@outlook.com (W.Y.); gaohao0302@outlook.com (H.G.); 2 Antai College of Economics and Management, Shanghai Jiao Tong University, Shanghai 200030, China; 3 Faculty of Finance, City University of Macau, Macau 999078, China; jiachengliao@outlook.com

**Keywords:** international trade, embodied carbon, carbon emission reduction, multi-region input-output analysis

## Abstract

With the rapid growth of China’s export trade and increasing pressure of domestic carbon emission reduction, the issue of carbon embodied in export trade has attracted increasing attention from academic circles. This paper has constructed a calculation model for embodied carbon in China’s export trade by using the multi-region input-output model and the international input-output data from the World Input-Output Database (WIOD) database in order to calculate the amount of embodied carbon. Our objective is to analyze the main source industry and specific sectors of embodied carbon in China’s export trade, and to provide a quantitative basis for emission reduction under the “carbon neutrality” strategy. The findings reveal that the embodied carbon in China’s export trade mainly comes from the secondary industry, which accounts for more than 90% of the total embodied carbon in export trade, while the proportions of embodied carbon in the primary industry and the tertiary industry are relatively low, about 1% and 5–7%, respectively. In terms of specific sectors, the crop and animal production and hunting sectors have the largest share (over 60%) of embodied carbon in the export trade of the primary industry; in the export trade of the secondary industry, the main sources of embodied carbon are the manufacturing sector and the power, gas, steam and air-conditioning supply sectors, respectively accounting for around 50% and 45% of the total embodied carbon in the export trade of the secondary industry; as for the tertiary industry, the transport and storage sectors have the largest share of embodied carbon in the export trade, which is around 70%. Based on the above research results, this paper has provided relevant policy recommendations, which are optimizing the export structure, improving the energy consumption structure and the carbon emissions trading system.

## 1. Introduction

The global economy has achieved rapid development with the wave of globalization [1,2,3]. However, at the same time, problems such as climate change and environmental deterioration caused by growing emissions of carbon dioxide and other greenhouse gases have become increasingly prominent [4,5,6]. In the research field of international trade, scholars such as Bodansky and Lawrence [7], McAuslanda and Millimet [8], and Shahbaz et al. [9] etc. have put forward different theories on the relationship between trade and the environment. On the one hand, some theories claim that international trade has resulted in the expansion of production and excessive consumption of resources, which has a negative impact on the environment [10,11,12,13]. On the other hand, some theories believe that international trade will lead to growth of income and accelerate the international transfer of advanced technology. Therefore, in the long term, international trade can contribute to a low-carbon economy and has a positive effect on the environment [14,15,16,17].

This paper has selected China, the largest developing country in the world, as the research object. Since China’s reform and opening up in the late 1970s, the extensive growth model has led to a continuous increase in carbon dioxide emissions, which has had a great negative impact on sustainable development and people’s health [18,19,20,21]. Therefore, this paper conducts research on the embodied carbon in China’s export trade, which could help achieve carbon emission reduction, and promote the green development of foreign trade in developing countries. Moreover, under the current global climate change framework agreement, the responsibilities of carbon emission reduction are based on the “Producer Country”, that is, whoever produces is responsible [22,23,24]. Therefore, the research on the calculation of embodied carbon in China’s export trade could also help clarify carbon emission responsibilities in the field of international trade and provide scientific basis for global sustainable development initiatives.

Based on the research of Minunno et al., (2021) [25], this paper defines embodied carbon as the sum of direct and indirect carbon dioxide emissions in the process of producing products and/or services. Here, the direct emissions refer to the carbon emissions caused by the direct use of energy or other direct actions in the production of final products [26,27], while the indirect emissions refer to the carbon emissions generated by the input of intermediate products in the production process [28,29]. 

The carbon embodied in trade refers to the country-specific separation of production behavior and consumption behavior due to international trade. Although commodities are exported to foreign countries for consumption through international trade, the carbon dioxide generated in the production process is entirely borne by the exporting country [30]. Therefore, trade-embodied carbon is essentially the amount of carbon emissions produced by a country in international trade in order to meet the needs of other countries [31,32,33]. When participating in international trade, China has borne a large amount of carbon emissions in order to meet the needs of foreign countries, which has a great negative impact on the environment [34,35,36]. Therefore, this paper focuses on the embodied carbon in China’s export trade.

The academic community has conducted numerous studies on the calculation of embodied carbon in export trade. In 1970, Leontief combined the input-output model with environmental variables to study the impact of human economic activities on the natural environment [37]. Since then, the input-output method has been widely applied in the field of economics and environmental research. The assumptions of the model have been continuously improved, and the compilation of input-output tables has also made great progress. In the field of trade embodied carbon, researchers originally used the single-region input-output models to measure and analyze the embodied carbon in trade [38,39]. This model treats all countries/regions outside the home country as a whole, and calculates the embodied carbon in trade between the home country and all the external countries/regions [40,41].

In terms of research methods, researchers generally use single-region input-output models for analysis. For example, Sun et al. used a single-region model to calculate and analyze the carbon footprint of India’s economic sectors from 1995 to 2009, and examined the changes in the carbon emission intensity of various sectors. The results show that from a production perspective, the most prominent “high-carbon” sectors are the electricity, gas and water supply sectors, which account for 60% of the total CO_2_ emissions in the secondary industry [42]. Qian et al. used the improved Randomized Adaptive Search (RAS) algorithm to convert China’s single-region input-output table into comparable prices, and calculated the embodied carbon intensity of export trade based on this single-region input-output table. The results show that the embodied carbon intensity of China’s exports is higher than that of domestic production. Therefore, the environmental cost to obtain unit profits is higher in foreign trade than in domestic production [43]. Hu et al. constructed a single-region non-competitive input-output table and studied China’s embodied carbon emissions in international trade and inter-provincial trade. They also found that the embodied carbon intensity in China’s export trade is greater than that in import trade, which is due to China’s disadvantaged position in foreign trade. China actually plays the role of a “pollution shelter” [44]. Qu et al. improved the traditional single-region input-output model. They combined the single-region input-output table with the optimized entropy model, and calculated China’s embodied carbon emissions in 2016. They found that the secondary industries, especially the energy and metal product manufacturing sectors, are still the main contributors to China’s carbon emissions, accounting for more than 70% of the total embodied carbon emissions, while the tertiary industry only generates about 10% of the embodied carbon emissions [45]. Liu used China’s single-region non-competitive input-output table to estimate the bilateral embodied carbon emissions between China and major trading partners such as the European Union, the United States and Japan. The finding was that the embodied carbon in China’s export trade has been overestimated [46]. 

As further studies are conducted in this field, the shortcoming of the single-region input-output model has become more obvious. This model treats all countries or regions outside the country as a whole [47,48]. Therefore, no matter what kind of assumption is made on the technology development level and/or carbon emission intensity of the import source, it cannot reflect the difference in the energy consumption structure and/or the development level of production technologies of different countries or regions, which will inevitably cause errors in the calculation results [49,50]. Considering this issue, the multi-region input-output model has been gradually recognized by the academic circle. This model abandons the assumption that the foreign production technology and carbon emission intensity are all the same, and calculates the import part of intermediate products and final products according to different source countries or regions, respectively. This model has fully considered the technological differences between different countries or regions, which makes the calculation result of embodied carbon in trade more accurate and reliable [51,52,53,54,55].

Due to the assumption of technological homogeneity of imported goods in the single-region input-output model, this model does not distinguish the origin of imported goods, and therefore may cause errors in the calculation results [56,57,58]. Therefore, this paper will adopt a multi-region input-output method to calculate the embodied carbon in trade. Our objective is to analyze the main source industry and specific sectors of embodied carbon in China’s export trade, and to provide a quantitative basis for emission reduction under the “carbon neutrality” strategy.

There are also some researches using the multi-region input-output method in the existing literature [59,60,61]. Compared with them, our study uses the WIOD data and conducts detailed calculations of the sources of embodied carbon in China’s export trade, achieving a more in-depth analysis.

We try to contribute to existing research in the following two ways:

(1) Based on the multi-region input-output method and WIOD data, we calculated the embodied carbon in the export trade of China, the world’s largest carbon dioxide emitter, which enriches the existing literature.

(2) The source analysis of embodied carbon in China’s export trade provides an important reference for the study of embodied carbon in trade in other countries in the world, especially developing countries. It also helps these countries to achieve emission reductions as soon as possible.

The structure of this paper is as follows: Section 2 introduces the specific research methods and data source, and constructs the estimation model for trade-embodied carbon; Section 3 calculates the embodied carbon emissions in China’s export trade, and analyzes the embodied carbon emissions of different industries; Section 4 provides conclusions for this paper, and corresponding policy recommendations.

## 2. Materials and Methods

### 2.1. Input-Output Analysis Method

The input-output table established by Leontief can accurately reflect the direct connections between various sectors in the economic system [62]. Therefore, it has been widely adopted to quantitatively analyze the economic relationship between input and output in the production activities of various sectors [63,64,65], in order to gain a deeper understanding of the operation patterns of the economic system. It is because of the above advantage of the input-output analysis method that it is widely used by the academic circle to calculate the value added and embodied carbon in trade activities with the existence of intermediate trade [66,67,68]. 

A country’s input-output table depicts the connections between various industries, products, and users within the country, and is also called a single-region input-output table [37]. In the context of globalization of the current economy and division of labor, the single-region input-output table cannot fully reflect the links in production, trade, and consumption between countries and economies [69]. Therefore, it is necessary to expand the regional dimension of this input-output table. After expansion of the regional dimension, the national input-output table has evolved into the international input-output table, which is also known as a multi-region input-output table [70,71]. This table can better illustrate the relationship between input and output of various economic sectors in different countries or regions, and has higher academic and application value in terms of study on international trade and operation patterns of the international economy. This paper first constructs an international multi-region input-output table as shown in Table 1:

where: 

$Z^{rs}$ is a matrix of intermediate products produced by country r for production in country s;

$Z_{ij}^{rs}$ represents the intermediate products that are produced by sector i of country r and used for production in sector j of country s;

$F^{rs}$ is the column vector for demand of country s for the final products produced by sectors in country r;

$F_{i}^{rs}$ represents the demand of country s for the final products produced by sector i of country r, including consumption demand and investment demand; 

$X^{r}$ is the column vector for total output of each sector in country r;

$X_{i}^{r}$ represents the total output of sector i in country r, which includes both intermediate products and final products; 

$I^{s}$ is the row vector for initial input of each sector in country s; 

$I_{j}^{s}$ represents the value of initial input invested by sector j of country s in production activities; 

$Y^{s}$ is the row vector for total input of country s;

$Y_{j}^{s}$ represents the total input of sector j in country s, which includes both the initial input in production activities of sector j and the intermediate input of other sectors. 

Generally speaking, the total production of a sector should be equal to the total input of this sector. 

Below Equation (1) shows the relationship in the rows of the input-output table:

(1) $$\underset{s}{\sum }\underset{j}{\sum }Z_{ij}^{rs}+\underset{s}{\sum }F_{i}^{rs}=X_{i}^{r}\;\left(r=1,2,\ldots ,M,i=1,2,\ldots ,N\right)$$

For each row, the sum of all the intermediate products and final products produced by one sector should be equal to the total output of this sector. In the above equation, M is the total number of economies and N represents the number of production sectors of each economy. 

Convert Equation (1) to a matrix format, as shown in Equation (2):

(2) Z+F=X

where Z is shown as Equation (3):

(3) $$Z=\left[\begin{array}{ccc} Z^{11} & \ldots & Z^{1M}\\ \ldots & \ldots & \ldots \\ Z^{M1} & \ldots & Z^{MM} \end{array}\right]$$

Z represents the combination of the intermediate product matrix between different countries, and similarly:

(4) $$F=\left[\begin{array}{ccc} F^{11} & \ldots & F^{1M}\\ \ldots & \ldots & \ldots \\ F^{M1} & \ldots & F^{MM} \end{array}\right]$$

(5) $$X=\left[\begin{array}{c} X^{1}\\ \ldots \\ X^{M} \end{array}\right]$$

Equations (4) and (5) give the final demand vector and the total output vector of each country, respectively, providing a basis for the calculation of the direct consumption coefficient. 

The direct consumption coefficient is one of the most important and basic concept in the input-output analysis [72]. It reflects the direct consumption of the products of other sectors in terms of the unit output of each production sector in production activities. It is the most direct manifestation of the economic links between various sectors in the economic system, and its calculation method is shown in Equation (6):

(6) $$A_{ij}^{rs}=\frac{Z_{ij}^{rs}}{Y_{j}^{s}}\left(r、s=1,2,\ldots ,M,i、j=1,2,\ldots ,N\right)$$

Because the total output of a sector is equal to its total input, that is, $Y_{j}^{s}=X_{j}^{s}$, the above equation can also be converted to Equation (7) below:

(7) $$A_{ij}^{rs}=\frac{Z_{ij}^{rs}}{X_{j}^{s}}\;\left(r、s=1,2,\ldots ,M,i、j=1,2,\ldots ,N\right)$$

where $A_{ij}^{rs}$ is the direct consumption coefficient. 

Therefore, the direct consumption coefficient can also be called the technical coefficient. Its value depends on the development level of technology and management, the product structure, and the price of intermediate products. The direct consumption coefficient can be further transformed to a matrix format, as shown in Equation (8):

(8) $$A=\left[\begin{array}{ccc} A^{11} & \ldots & A^{1M}\\ \ldots & \ldots & \ldots \\ A^{M1} & \ldots & A^{MM} \end{array}\right]$$

The above equation can be written as Equation (9) below:

(9) AX+F=X

After the inverse operation of the matrix, Equation (10) can be obtained:

(10) $$X={\left(I-A\right)}^{-1}F$$

Equation (10) describes the relationship between the final demand and the total output of various sectors of each economy, in which ${\left(I-A\right)}^{-1}$ is the Leontief inverse matrix, also known as the total demand coefficient matrix. The total demand coefficient is relative to the direct consumption coefficient. Due to the complexity of economic activities, in a production activity, the consumption of products from one sector by another sector is often not limited to the direct consumption of products or services, but also covers the indirect consumption of products or services. Because the intermediate products consumed during production activities often include the consumption by other sectors, the direct consumption coefficient cannot reflect the indirect consumption of this sector. Take the automobile sector as an example. In the process of car manufacturing, electricity is directly consumed. Such direct consumption can be reflected by the direct consumption coefficient. However, in the car manufacturing process, the production of the intermediate products, such as tires, steel, etc. also consumes electricity. This part is the indirect consumption of electricity, which cannot be reflected by the direct consumption coefficient. Therefore, by calculating the total demand coefficient, we can obtain the total input required by each sector to produce each unit of the final product, and theoretically use the Leontief model to describe the intricate relationship between the final demand and the total output of each sector, which has significant implications for studies. 

### 2.2. Calculation Model of Trade Embodied Carbon

The input-output analysis methods also play an important role in studying environmental issues. By linking with the environmental account, this method can measure the flow of trade-embodied carbon, and can measure the carbon emissions produced by one country that is driven by the demand of another country. The specific calculation method is as follows:

First, obtain the carbon emission data of various countries and various sectors from the environmental account, and calculate the direct carbon emission coefficient, as shown in Equation (11):

(11) $$c_{i}^{r}=\frac{CO_{2i}^{r}}{X_{i}^{r}}\;\left(r=1,2,\ldots ,M,i=1,2,\ldots ,N\right)$$

where $CO_{2i}^{r}$ is the carbon dioxide emissions of sector i in country r, and $c_{i}^{r}$ is the direct carbon dioxide emission coefficient of sector i in country r, which represents the amount of carbon dioxide emissions of sector i in country r per unit of total output. The direct carbon dioxide emission coefficient can be written as a matrix, as shown in Equation (12):

(12) $$c=\left[\begin{array}{c} c^{1}\\ \ldots \\ c^{M} \end{array}\right]$$

By linking Equation (12) with the Leontief model, we can obtain Equation (13):

(13) $$c⨀X=c⨀{\left(I-A\right)}^{-1}F$$

where c⨀X represents the vector obtained by multiplying the corresponding positions of vector c and vector X, and $c⨀{\left(I-A\right)}^{-1}$ is the total carbon emission coefficient, which represents the total carbon emissions generated from each unit of final product produced by each sector in each country. Here, the final demand matrix F can be expressed as a block matrix for illustration, as shown in Equation (14):

(14) $$F=\left[\begin{array}{ccc} F^{11} & \ldots & F^{1M}\\ \ldots & \ldots & \ldots \\ F^{M1} & \ldots & F^{MM} \end{array}\right]=\left[\begin{array}{ccc} F^{1} & \ldots & F^{M} \end{array}\right]$$

The final demand matrix F is divided by columns into demand vectors for the final product of each country and each sector, where $F^{s}$ is a $\left(M*N\right)\times 1$ dimensional vector, representing the demand of country s for the final product of each country and each sector. Thus, the calculation method of trade embodied carbon can be obtained, as shown in Equation (15):

(15) $$\begin{array}{c} C=c⨀{\left(I-A\right)}^{-1}F\\ =\left[c⨀{\left(I-A\right)}^{-1}F^{1}\;\ldots \;c⨀{\left(I-A\right)}^{-1}F^{s}\;\ldots \;c⨀{\left(I-A\right)}^{-1}F^{M}\right] \end{array}$$

In the above equation, $c⨀{\left(I-A\right)}^{-1}F^{s}$ represents the total embodied carbon produced by various countries and sectors in order to meet the final demand of country s. By further dividing the matrix by countries, we can obtain Equation (16) as below:

(16) $$C=\left[\begin{array}{ccc} C^{11} & \ldots & C^{1M}\\ \ldots & C^{rs} & \ldots \\ C^{M1} & \ldots & C^{MM} \end{array}\right]$$

where $C^{rs}$ is the vector of carbon dioxide emissions generated from production activities of various sectors in country r in order to meet the final demand of country s. If r≠s, $C^{rs}$ is the embodied carbon export transferred from country r to country s through trade; if r=s, $C^{rs}$ is the carbon emissions generated from one country’s production activities to meet its own demands. Thus, the embodied carbon export vector of country r can be obtained as shown in Equation (17):

(17) $$EC^{r}=\underset{s,s\neq r}{\sum }C^{rs}$$

In Equation (17), $EC^{r}$ is an N×1. dimensional vector, representing the embodied carbon in the export trade of various sectors in country r.

### 2.3. Data Source and Calculation Process

Currently, the majority of international input-output tables are from the World Input-Output Database (WIOD) [73], Organization for Economic Cooperation and Development (OECD) [74], Global Trade Analysis Project (GATP) [75], Asian Development Bank (ADB) [76] and Eora databases [77]. This paper has used the WIOD input-output tables as the data source, with the following reasons. First, the WIOD’s international input-output table has subdivided exports into three categories: intermediate demand, final consumption, and final investment, and each category is broken down by source country using a fixed row ratio. Therefore, its data are more precise and accurate than other databases. Second, the WIOD database has supporting economic and social accounts as well as environmental accounts. It is relatively more complete, which is convenient for extended research.

The WIOD database has two versions, the 2013 version and the 2016 version. This paper uses the 2016 version [78], which includes complete data of 56 economic sectors and 43 countries and regions, covering 28 EU countries and 15 other countries and regions. It provides three formats (Stata, R and excel) for download [78]. In the database, the economic sectors are mainly classified according to the latest International Standard Industry Classification System (ISIC Rev 4.0), covering time series data from 2000 to 2014.

In the supporting environmental accounts, the carbon emission data are only updated to 2011. Therefore, the carbon emission data in this paper are from the environmental accounts from 2000 to 2016 published by the European Commission Joint Research Center in 2019 [79], which includes the energy consumption and carbon emission data of 56 sectors in 44 economies, in which the division of economies and sectors is the same as that of WIOD’s international input-output table.

## 3. Results and Discussion

### 3.1. Analysis of the Calculation Results of the Total Embodied Carbon in China’s Export Trade

**Figure 1:**
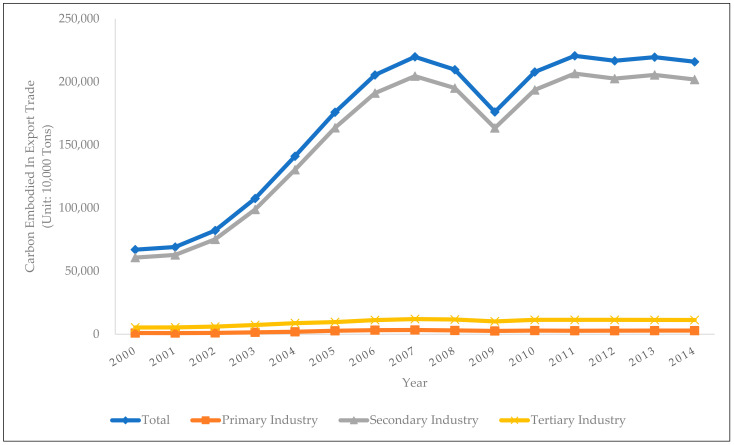
China’s Export Trade Embodied Carbon and Its Distribution Across Three Main Industries.

Based on the aforementioned international input-output table and input-output analysis method, as well as the WIOD database, this paper has calculated the embodied carbon in China’s export trade and industrial export trade, as shown in Figure 1:

In terms of the time trends, China’s overall embodied carbon in export trade showed a rapid growth trend from 2000 to 2007, i.e., from 670 million tons in 2000 to 2.20 billion tons in 2007. The embodied carbon started to decline after 2007 and reached a local lowest level of 1.76 billion tons in 2009. However, after that, it rebounded to around 2.1 billion tons and remained at this level. Among the three main industries, the primary industry showed a similar trend to the overall trend in terms of the embodied carbon in export trade. Its embodied carbon increased from 9.33 million tons in 2000 to 33.32 million tons in 2007. After that, the embodied carbon also experienced some decline with a rebound afterwards, and then remained at the level of about 27 million tons. As for the secondary industry, its embodied carbon in export trade has shown a highly consistent trend with the embodied carbon in the overall export trade, which increased from 607 million tons in 2000 to 2.04 billion tons in 2007, and then fell to 1.63 billion tons in 2009, and then rebounded and remained at a level of 2 billion tons. The trend of the export trade-embodied carbon in the tertiary industry is basically the same as the other two industries, which increased from 53.55 million tons in 2000 to 119 million tons in 2007, and then dropped to 101 million tons, and then rebounded to 110 million tons and stayed at that level.

In terms of the horizontal comparison across the industries, the secondary industry is the most important source of embodied carbon in China’s overall export trade. The export trade-embodied carbon of the secondary industry accounts for more than 90% of the embodied carbon in the overall export trade, while the corresponding proportions of the primary industry and the tertiary industry are only about 1% and 5–7%, which are far lower than that of the secondary industry.

In summary, with the rapid growth of China’s foreign trade, the environmental costs are also increasing. Since 2000, the export trade-embodied carbon of China’s three main industries has increased, and the overall embodied carbon in export trade has reached 2.1 billion tons. As far as the industrial structure is concerned, the embodied carbon generated by the secondary industry is the most important source of embodied carbon in China’s export trade.

### 3.2. Analysis of Embodied Carbon in Export Trade of the Primary Industry

**Figure 2:**
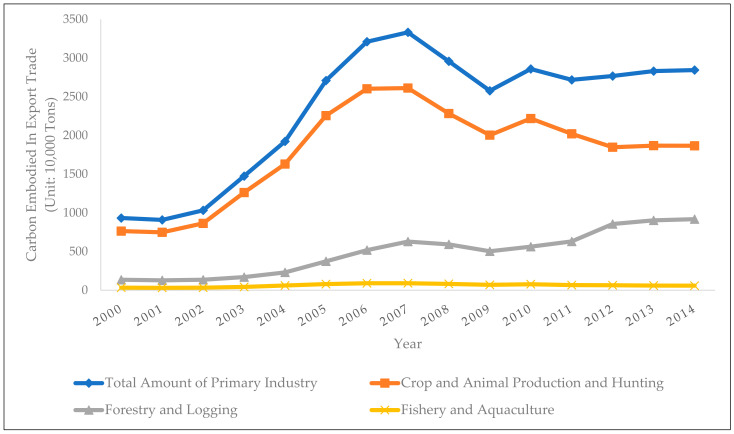
Embodied Carbon in the Export Trade of China’s Primary Industry.

Using the aforementioned international input-output table and input-output analysis method, as well as the WIOD database, this paper has calculated the embodied carbon in the export trade of China’s primary industry, as shown in Figure 2:

In terms of the time trends, the embodied carbon in the export trade of the crop and animal production and hunting sectors has experienced a huge increase from 2000 to 2007, which increased from 7.64 million tons in 2000 to 26.02 million tons in 2007, reaching the peak level across the years. After that, it has shown a slow declining trend, and reached a local minimum of 20.03 million tons in 2009. By 2014, the embodied carbon in export trade of the crop and animal production and hunting sectors has reached 18.66 million tons. The embodied carbon in export trade of the forestry and logging sectors has shown a slow growth trend over the years, which has increased from 1.35 million tons in 2000 to a local peak of 6.29 million tons in 2007. After that, it further increased after a short decline. In 2014, the embodied carbon of export trade in the forestry and logging sectors was 9.19 million tons. The embodied carbon in the export trade of the fishery and aquaculture sectors has been relatively stable from 2000 to 2014. It has decreased slowly after a slow growth, increasing from 336.6 thousand tons in 2000 to its peak of 889.8 thousand tons in 2006, with continuous declination afterwards. In 2014, the embodied carbon of export trade in the fisheries and aquaculture sectors was 588.4 thousand tons.

In terms of horizontal comparison across sectors, the embodied carbon in the export trade of the primary industry mainly comes from the crop and animal production and hunting sectors. From 2000 to 2006, the embodied carbon of this sector accounted for more than 80% of the entire primary industry. After that, the proportion has decreased, but has still remained above 60%. Meanwhile, the proportion of the embodied carbon of the forestry and logging sectors has gradually increased from 15% to more than 30%. The fisheries and aquaculture sectors have the lowest proportion of embodied carbon in the total export trade of the primary industry, which has remained below 4%.

### 3.3. Analysis of Embodied Carbon in Export Trade of the Secondary Industry

**Figure 3:**
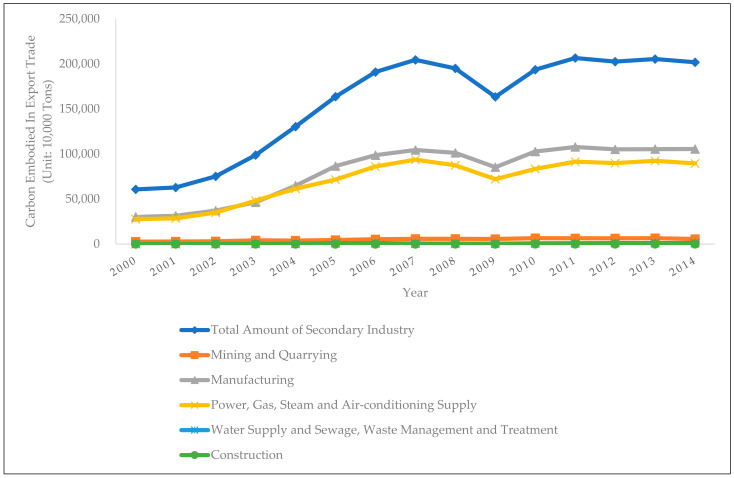
Embodied Carbon in the Export Trade of China’s Secondary Industry.

Using the aforementioned international input-output table and input-output analysis method, as well as the WIOD database, this paper has calculated the embodied carbon in the export trade of China’s secondary industry, as shown in Figure 3:

In terms of the time trends, the embodied carbon in export trade of the mining and quarrying sectors has showed an upward trend from 2000 to 2010, increasing from 27.08 million tons in 2000 to 65.51 million tons in 2010, with a slight downward trend thereafter. In 2014, the embodied carbon of export trade in the mining and quarrying sectors was 55.91 million tons. The embodied carbon in the export trade of the manufacturing sector has showed a substantial increase from 2000 to 2009, increasing from 301 million tons in 2000 to 1.04 billion tons, with a short decline thereafter. In 2009, it declined to a local minimum of 852 million tons, and then rebounded to around 1 billion tons, with small fluctuations within this range. The overall trend of embodied carbon of the power, gas, steam and air-conditioning supply sectors is the same as that of the manufacturing sector. It has shown an upward trend from 2000 to 2007, reaching 937 million tons in 2007, and then declined to a local minimum of 720 million tons in 2009. It has risen to around 900 million tons since then and fluctuated around that level. The embodied carbon in export trade of the water supply and sewage, waste management and treatment sectors has shown a slow upward trend, growing from 10.35 million tons in 2000 to 11.28 million tons in 2014. The embodied carbon in the export trade of the construction sector has also showed a slight upward trend between 2000 and 2014, slowly rising from 273 thousand tons in 2000 to 843.2 thousand tons in 2014.

In terms of horizontal comparison across sectors, the embodied carbon in the export trade of the secondary industry mainly comes from the manufacturing sector and the power, gas, steam and air-conditioning supply sectors, which, respectively, accounted for around 50% and 45% of the total embodied carbon in the export trade of the secondary industry. The proportion of the mining and quarrying sectors fluctuated between 2% and 5%; while those of the construction sector and the water supply and sewage, waste management and treatment sectors were relatively small, both below 1%.

### 3.4. Analysis of Embodied Carbon in Export Trade of the Tertiary Industry

**Figure 4:**
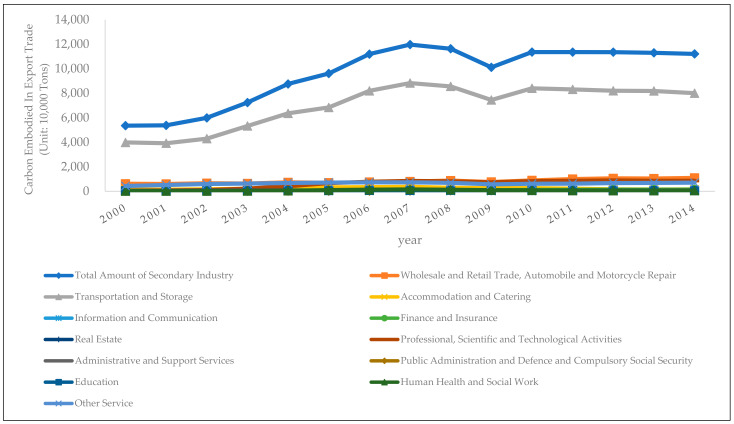
Embodied Carbon in the Export Trade of China’s Tertiary Industry.

Using the aforementioned international input-output table and input-output analysis method, as well as the WIOD database, this paper has calculated the embodied carbon in the export trade of China’s tertiary industry, as shown in Figure 4:

In terms of the time trends, the embodied carbon in export trade of the wholesale and retail trade sector and the automobile and motorcycle repair sector has shown a slight upward trend from 2000 to 2014, rising from 6.12 million tons in 2000 to 10.89 million tons in 2014. The embodied carbon of export trade in the transportation and storage sectors has shown a strong upward trend in the early stage, growing from 39.84 million tons in 2000 to its peak of 88.28 million tons in 2007. After that, it has shown a downward trend, reaching a local minimum of 74.48 million tons in 2009, and then rebounded to 80.01 million tons in 2014. The embodied carbon of the accommodation and catering sectors has experienced a slow increase and a slow decline between 2000 and 2014. It has risen from 1.27 million tons in 2000 to 3.61 million tons, and then dropped to 1.51 million tons in 2014. The embodied carbon of the professional, scientific and technological activities sector has showed an upward trend in the early stage, rising from 590.6 thousand tons in 2000 to 8.43 million tons in 2007, and remained at around 8 million tons since then. Because the export trade-embodied carbon of other sectors is relatively small, this paper will not elaborate on that.

In terms of horizontal comparison across sectors, the embodied carbon in the export trade of the tertiary industry mainly comes from the transportation and storage sectors, with a proportion of over 70%. The proportion of the wholesale and retail trade sector and the automobile and motorcycle repair sector is around 10%; that of the accommodation and catering sectors is around 2%; that of the professional, scientific and technological activities sector was 1.1% in 2000, which further rose to around 7% thereafter.

## 4. Conclusions and Policy Recommendations

Due to the rapid growth of China’s export trade and the increasing pressure of carbon emission reduction, the topic of embodied carbon in export trade has attracted increasing attention from academic circles. In view of this, this paper has constructed a calculation model for embodied carbon in China’s export trade by using the multi-region input-output model and the international input-output data from the WIOD database in order to calculate the amount of embodied carbon. Through calculation and analysis, this paper has found that the embodied carbon in China’s export trade mainly comes from the secondary industry, which accounts for more than 90% of the total embodied carbon in export trade, while the proportions of embodied carbon in the primary industry and the tertiary industry are relatively low, about 1% and 5–7%, respectively. In terms of specific sectors, the crop and animal production and hunting sectors have the largest share (over 60%) of embodied carbon in the export trade of the primary industry; in the export trade of the secondary industry, the main sources of embodied carbon are the manufacturing sector and the power, gas, steam and air-conditioning supply sectors, respectively, accounting for around 50% and 45% of the total embodied carbon in the export trade of the secondary industry; as for the tertiary industry, the transport and storage sectors have the largest share of embodied carbon in the export trade, which is around 70%.

Therefore, this paper proposes the following policy recommendations:

First, optimize the export structure. It is recommended to adjust the export structure of the three industries of the national economy, and enhance the export trade of the tertiary industry, especially the modern service industry. According to the findings above, the embodied carbon intensity of the export trade of China’s tertiary industry is relatively low, and the environmental cost of earning unit profit is also lower for the tertiary industry. Therefore, vigorously enhancing the export trade of the tertiary industry, especially the modern service industry, will help reduce the total embodied carbon in China’s export trade. In addition, because the secondary industry is the main source of embodied carbon in China’s export trade, the optimization of the structure of the secondary industry and the development of production capacity of high value-added and low energy consumption export commodities can help improve the export structure and reduce the overall embodied carbon in China’s export trade.

Second, improve the energy consumption structure. China is a country rich in coal but relatively scarce in oil and natural gas, so coal is still the dominant energy source in the current primary energy consumption structure [80,81,82]. In the process of providing export products and services, the large-scale consumption of coal by various industries has led to high carbon emissions. Therefore, there is an urgent need to improve the energy consumption structure. Although the energy structure with coal and other fossil energy being the main energy source cannot be changed in a short period of time, it is still possible to develop clean production technologies in fields such as supercritical coal-fired power generation and coal gasification, etc. by upgrading technologies in order to strengthen clean production in the export sector [83,84]. At the same time, it is necessary to reduce the proportion of coal in the primary energy consumption structure as much as possible. By increasing the proportion of natural gas in the primary energy structure, developing safe nuclear power, vigorously introducing clean and renewable energy such as hydropower, wind power, solar energy, geothermal energy, and biomass energy, we can effectively improve the energy consumption structure in export trade and reduce the embodied carbon in export trade [85,86].

Finally, improve the carbon emissions trading system. According to the “United Nations Framework Convention on Climate Change” [87] and the additional “Kyoto Protocol” [88] adopted by the United Nations Panel on Climate Change, it is a practical international policy tool to resolve excessive emissions of greenhouse gases through a market mechanism. Therefore, China should also actively participate. By establishing a modern carbon emissions trading market and allowing export enterprises to trade carbon emissions as a commodity with each other, we can reduce the embodied carbon in export trade. In terms of specific policy measures, carbon emissions trading first needs to set carbon emission quotas for each export enterprise, and companies with surplus emissions can take the excess emissions to the market and sell them to other companies with insufficient emissions. By establishing and improving the carbon emissions market and trading system, we can fully utilize the market mechanism to turn the positive externalities of companies with surplus emissions into profits, and turn the negative externalities of companies with insufficient emissions into costs in order to effectively control the carbon emissions of export enterprises. In 2013, Shenzhen took the lead in establishing a carbon emissions trading platform as a pilot project [89]. There are seven cities and/or provinces in China that have launched pilot carbon emissions trading platforms—Shenzhen, Beijing, Tianjin, Shanghai, Guangdong, Hubei, and Chongqing [90], which could play a positive role in reducing the embodied carbon in export trade.

## Figures and Tables

**Table 1:** International Multi-Region Input-Output Table.

	Output	Intermediate Demand				Final Demand				Total Output
Input		1	2	…	*M*	1	2	…	*M*	
Intermediate Input	1	*Z* ^11^	*Z* ^12^	…	*Z* ^1*M*^	*F* ^11^	*F* ^12^	…	*F* ^1*M*^	*X* ^1^
	2	*Z* ^21^	*Z* ^22^	…	*Z* ^2*M*^	*F* ^21^	*F* ^22^	…	*F* ^2*M*^	*X* ^2^
	…	…	…	…	…	…	…	…	…	…
	*M*	*Z^M^* ^1^	*Z^M^* ^2^	…	*Z^MM^*	*F^M^* ^1^	*F^M^* ^2^	…	*F^MM^*	*X^M^*
Initial Input		*I* ^1^	*I* ^2^	…	*I^M^*	_	_	_	_	_
Total Input		*Y* ^1^	*Y* ^2^	…	*Y^M^*	_	_	_	_	_

## Data Availability

The calculation data used in this paper come from the WIOD database, and the environmental accounts published by the European Commission Joint Research Center, which have been explained in the main text.
